# Personalized machine learning models for noninvasive hypoglycemia detection in people with type 1 diabetes using a smartwatch: *Insights into feature importance during waking and sleeping times*

**DOI:** 10.1371/journal.pone.0325956

**Published:** 2025-06-25

**Authors:** Yasmine M. Mohamed, José Mancera, Andreas Brandenberg, Stefan Fischli, Michael M. Havranek

**Affiliations:** 1 Lucerne University of Applied Sciences and Arts, Lucerne, Switzerland; 2 Division of Endocrinology, Diabetes and Clinical Nutrition, Lucerne Cantonal Hospital, Lucerne, Switzerland; 3 Competence Center for Health Data Science, Faculty of Health Sciences and Medicine, University of Lucerne, Lucerne, Switzerland; Japan Medical Exercise Association, JAPAN

## Abstract

Hypoglycemia is a major challenge for people with diabetes. Therefore, glycemic monitoring is an important aspect of diabetes management. However, current methods such as finger pricking and continuous glucose monitoring systems (CGMS) are invasive, and hypoglycemia has still been shown to occur despite advancements in CGMS. Consequently, a growing body of research has been directed toward noninvasive hypoglycemia detection, relying on data from medical devices and wearables that can record physiological changes elicited by hypoglycemia. Consumer-grade wearables such as smartwatches remain an attractive yet underexplored candidate for such applications. Therefore, we explored the potential of a consumer-grade wearable for hypoglycemia prediction and investigated differing feature importance during waking and sleeping times. Smartwatch data from 18 adults with type 1 diabetes was collected, preprocessed, and imputed. Machine learning (ML) models were built using a tree-based ensemble algorithm to detect hypoglycemic events registered by CGMS. Models were built in a personalized manner using the same participant’s data for training and testing, with separate modeling for daytime and nighttime. The relative importance of input features on model decisions was analyzed using SHAP (SHapley Additive exPlanations). Seventeen personalized models were built with an average area under the receiver operating characteristic curve (AUROC) score of 0.74 ± 0.08. Average specificity and sensitivity were 0.76 ± 0.18 and 0.71 ± 0.15, respectively. Time-of-day, activity, and cardiac features showed comparable importance in daytime models (29.9%, 28.5%, and 24%, respectively), while in nighttime models, cardiac features demonstrated the highest importance (42.2%) followed by time-of-day features (37.5%) and respiratory features (15.2%). In summary, we demonstrate the potential of consumer-grade wearables in noninvasive hypoglycemia detection. By additionally considering different physiological states (waking and sleeping) during modeling, our results offer further insights into differences in relative feature importance influencing the model’s decision, guiding future research in this area.

## Introduction

Diabetes is one of the most common chronic diseases worldwide [1]. To date, it has no cure and is managed by maintaining a balance of diet, exercise, and medication [2]. In type 1 diabetes, hypoglycemic episodes (i.e., low blood glucose level) are a major challenge in disease management and have been associated with increased morbidity (through falls, road accidents, etc.) and mortality [3,4]. Hypoglycemia additionally imposes a psychological burden due to its unpredictable nature and the related feelings of fear, affecting quality of life [3,5]. Therefore, glycemic monitoring is an important aspect of diabetes management. Invasive methods such as finger pricking and continuous glucose monitoring systems (CGMS) are currently utilized for this purpose, and while CGMS have improved glycemic monitoring by providing real-time feedback [6], recent reports have shown that hypoglycemic events are still experienced despite their use [7,8]. This highlights the need for additional supportive tools for diabetes management. Thus, a growing body of research is aiming to develop noninvasive alternatives for hypoglycemia detection, due to the immediate risks it poses.

Importantly, hypoglycemia is known to elicit changes in physiological parameters, such that several studies have attempted to investigate and leverage these changes to noninvasively predict hypoglycemia occurrence. The effect of hypoglycemia on cardiac parameters, such as heart rate and heart rate variability (HRV), has been a major focus of this approach [9–16]. For example, Olde Bekkink et al. [9] investigated changes in HRV due to hypoglycemia using electrocardiogram (ECG) data collected by a wearable medical patch, finding varied HRV patterns in response to hypoglycemia among individuals. Another study by Fellah Arbi et al. [10] trained recurrent convolutional neural networks to determine blood glucose levels based on ECG data collected using a wearable medical patch. More recently, Dave et al. [11] reported the development of personalized fusion models and achieved area under the curve values of 0.75 for detecting hypoglycemia and 0.78 for hyperglycemia, using ECG signals obtained via a chest strap.

However, the medical devices and wearables (e.g., ECG equipment, chest strap/patch) employed for data collection in these studies are either inapplicable or impractical in real-life settings. Consumer-grade wearables (such as smartwatches) are promising alternatives but remain underexplored in this context since they are generally perceived as less reliable [17], despite their many attractive features such as practicality, ubiquity, and usability. To our knowledge, only one previous study has attempted to use physiological data from consumer-grade wearables for noninvasive hypoglycemia detection, by combining two different wrist-worn wearables. Data from 22 participants was collected over 30 days and used to train a decision tree-based machine learning model that detected hypoglycemia with an area under the receiver operating characteristic curve (AUROC) score of 0.76 using 37 input features, categorized into cardiac, motion, electrodermal activity, and time features [18].

While time-related features were incorporated as an input to the model, no distinction was made between data collected during waking and sleeping times during modeling. Cross-subject validation was employed, wherein data from one participant was used for training the model while testing and validation was performed on data from another paired participant. This approach and others such as leave-one-out cross-validation, where data from all participants but one is used for model training while testing is performed on the left-out participant, allow for more reliable evaluation of model performance by avoiding possible data leakage. However, they might not be readily implementable in practice as they necessitate continuous model calibration across geographical regions and populations to account for interpersonal differences and allow generalizability, which is difficult to maintain as it requires ongoing labeled data that is not easily obtainable in medical contexts.

In this study, we investigated the potential of using a commercially available smartwatch for noninvasive detection of hypoglycemia using a combination of physiological and motion data. We distinguished between waking and sleeping times by creating separate machine learning models to account for important variations in physiological parameters between these two states [19–21]. This methodology allowed us to incorporate input features based on their relevance to the time of day (e.g., physical activity is more relevant during the day). Analysis of the relative importance of input features provided insights into the differential influence of these features on model decisions during waking and sleeping times. Moreover, we employed a custom modeling approach that enabled building personalized models for each participant using their own data; this strategy addressed the challenges of using same-participant time series data where the risk of data leakage is a concern. As such, our personalized approach facilitates more practicable real-life implementation (e.g., in the form of a smartwatch application) while circumventing the pitfalls of the aforementioned approaches.

## Materials and methods

### Study design and population

Eighteen adults with type 1 diabetes (12 males, 6 females, aged 36.7 ± 13.2 years) were recruited in the endocrinology and diabetology division at the Lucerne Cantonal Hospital between May 2023 and June 2024. Participants were using multiple daily injections (MDI) or conventional subcutaneous insulin infusion (CSII) without a hybrid closed-loop system. Exclusion criteria included having comorbidities, use of medication affecting the heart rate, severe hypoglycemia unawareness (Clarke score ≥4), and regular strenuous physical activity. Full inclusion and exclusion criteria are listed in S1 Table. The study was approved by the Ethics Committee of Northwest and Central Switzerland (EKNZ 2023−00229). All methods in this study were performed in accordance with the relevant guidelines and regulations, and all participants provided written informed consent.

### Procedure and data collection

Participants were provided with a blinded continuous glucose monitor (Dexcom G6) to record hypoglycemia outcomes and a smartwatch (Apple Watch Series 8) to collect the predictor variables consisting of heart rate (HR), heart rate variability (HRV), step count, energy expenditure, blood oxygen saturation (BOS), respiratory rate (RR), and time of day. Measurement intervals of the predictor variables were dictated by the Apple Watch itself. In addition, participants were asked to routinely measure their capillary blood glucose at least four times per day, as well as in cases of suspected hypoglycemia for validation purposes. Other CGMS apart from the blinded system were not permitted. Participants were given diaries to manually record information about mealtimes, meal content, insulin usage, capillary glucose measurements, and sleeping times. Data was collected for 10 days (i.e., the maximum duration of data collection by Dexcom G6), during which time participants carried out their normal daily activities. Data from all sources were merged based on timestamps, with each data point representing 1 minute.

### Outcome and predictors

Data collected from the CGMS was used to label the dataset with an outcome variable annotated as “1” when glucose levels dropped to <4 mmol/L for at least 15 minutes, and “0” otherwise. Table 1 summarizes the parameters collected from all data sources, including frequency of measurement, imputation methods, and engineered features. Fifteen input variables were derived in total: 13 from smartwatch data and two from manually documented data (i.e., mealtimes and short-acting insulin usage). Data imputation was performed by backfilling CGMS data for up to 5 minutes and forward filling HRV, RR, and BOS based on measurement frequency to avoid the possibility of data leakage from future data points.

**Table 1:** Data sources, imputation methods, and engineered features.

Data source	Feature	Frequency of measurement	Imputation method	Engineered features
CGMS^*^	Interstitial fluid glucose level	Every 5 min	Backfilling (limited to 5 min)	Outcome variable: “1” for hypoglycemic events lasting ≥15 min and “0” otherwise
Apple Watch	Heart rate (beats per minute)	Every min	–	• 10 min rolling average • 30 min rolling average • 60 min rolling average
	Heart rate variability (ms)	During the day: every 15–30 min, *only in restful states* During the night: every 15 min	Forward filling of last measured value (limited to 15 min)	• Last measured value • Difference from past measurement (if available)
	Blood oxygen saturation (%)	Every 30 min	Forward filling of last measured value (limited to 30 min)	Last measured value
	Respiratory rate (count per minute)	Every 15 min	Forward filling of last measured value (limited to 15 min)	Last measured value
	Step count (count per minute)	Every 1 min	–	30 min rolling sum 60 min rolling sum
	Active energy expenditure (kJ)	Every 1 min	–	30 min rolling sum 60 min rolling sum
	Time of day	–	–	Sine and cosine transformations of the hour of day
Manual	Mealtimes	Manually documented	–	Time since last meal
	Short-acting insulin	Manually documented	–	Insulin on board

* Continuous glucose monitoring systems.

Input features were derived either by direct usage of imputed features or by feature engineering (e.g., lagging features such as rolling averages; see Table 1). An additional HRV feature tracked the difference from the previous measurement, if available. Time-of-day features were derived from sine and cosine transformations of the hour of day to capture the cyclic nature of time.

Manually documented data (mealtimes and short-acting insulin usage) were simply recorded as “1” regardless of composition or dosage. Time since the last meal was added as an ordinal feature and insulin on board was estimated at 100%, 75%, 50%, and 25% over the 4 hours following short-term insulin usage. The manually-documented mealtimes and insulin usage were deliberately included in this simplistic manner in the modeling to enable a potential practical implementation in the future (e.g., in the form of a smartwatch application, see also the discussion section).

### Model building, evaluation, and interpretability

Binary classification models were built in Python (version 3.9.13) using XGBoost (eXtreme Gradient Boosting), a tree-based ensemble machine learning algorithm, owing to its effectiveness and ability to handle missing values [22]. Model output was the ability to detect the positive class, i.e., occurrence of hypoglycemia. Data were split using a custom method wherein days/nights with hypoglycemic events were iteratively selected as the test dataset, while the remaining data comprised the training dataset (see next section for more details). To overcome the large imbalance in the dataset, random undersampling was applied to the training dataset, while the test dataset remained imbalanced. Undersampling was preferred to oversampling techniques such as SMOTE (synthetic minority over-sampling technique) to avoid disrupting the temporal order of the data and training with redundant data points, possibly leading to model overfitting. Modeling was repeated using 10 different random states for undersampling to ensure reproducibility. Data was standardized prior to model training. Model hyperparameters were tuned to avoid overfitting by setting the L1 and L2 regularization terms to 10.

Waking and asleep states were modeled separately due to differentially available features (e.g., step count is irrelevant during sleep) and differences in physiological ranges for some variables (such as HR and HRV). Moreover, some parameters were exclusively measured during the night (e.g., RR and BOS). For prolonged nocturnal hypoglycemic events (n = 3), a cutoff value of 180 minutes was applied. Two participants (P03, P13) with incomplete meal records were modeled without mealtime-derived features.

**Fig 1:**
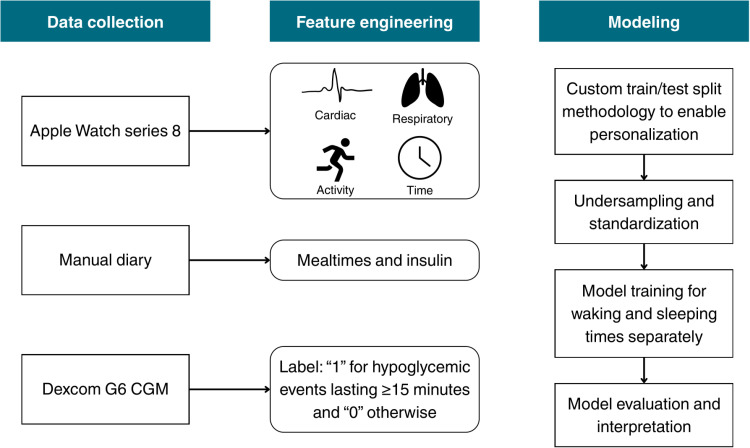
An overview of the methodology steps.

Model performance was evaluated using the AUROC value. Sensitivity and specificity were calculated using the optimal threshold identified via Youden’s J statistic [23], while the ability of the model to predict a hypoglycemic event within the first 15 minutes was also assessed. Model interpretability was derived by analysis of SHAP (SHapley Additive exPlanations) values to demonstrate the impact of different features on the model’s output [24]. The code used in this study is available on GitHub (https://github.com/YMM-0/smartwatch_hypo_detection.git) and the data on Zenodo (https://zenodo.org/records/15309060). Fig 1 shows an overview of the information provided here in the methodology section.

### Custom train/test split

We devised a custom methodology to enable building personalized models using data from the same patient for training and testing. Due to the timeseries nature of the data, the rows of the dataset are not i.i.d. (independently and identically distributed), therefore splitting the dataset randomly would lead to data leakage and an over-performing model. Ideally, a time series (or temporal) split is the method of choice; however, due to the uncertainty of hypoglycemia occurring toward the end of the participation period, strictly splitting the dataset on a temporal basis was rendered unfeasible. Instead, splitting the dataset was performed using a custom methodology.

**Fig 2:**
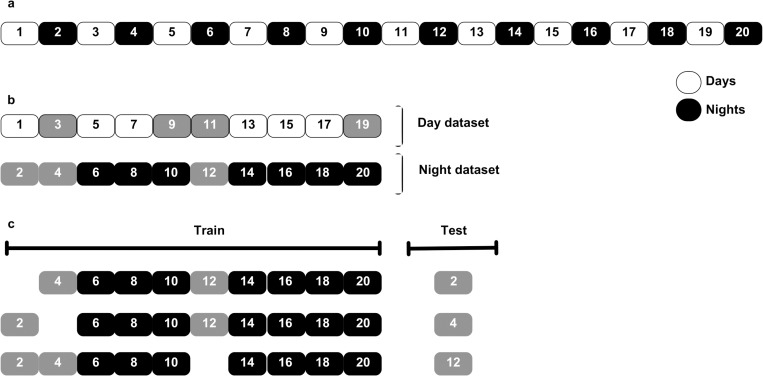
Shown is the custom train/test split methodology. a. Each day/night is given a unique identifier. b. The dataset is divided into “day” and “night” datasets. Days/nights where one or more hypoglycemic events were experienced by the participant are identified (highlighted in grey). c. Considering the night dataset to illustrate our approach, the custom training/test split is performed iteratively and each night where hypoglycemia occurred is singled out as the test dataset; the remaining days/nights comprise the training dataset.

Each day/night in the dataset was given a unique identifier as defined by the participant’s waking and sleeping times. The dataset was then divided into a so-called “day” dataset comprising days only and a “night” dataset comprising nights only. Days/nights where one or more hypoglycemic events occurred were identified. In an iterative process, the dataset was split so that a day/night where hypoglycemia occurred was singled out as the test dataset and the remaining days/nights comprised the training dataset, as depicted in Fig 2. It is important to note that the test dataset of one iteration was used to evaluate the model performance of that iteration only, in order to avoid reporting over-performing results from learning across iterations. The results from all iterations were finally averaged across iterations to provide the average result of the individualized prediction model of that participant.

## Results

### Recruitment and population characteristics

**Fig 3:**
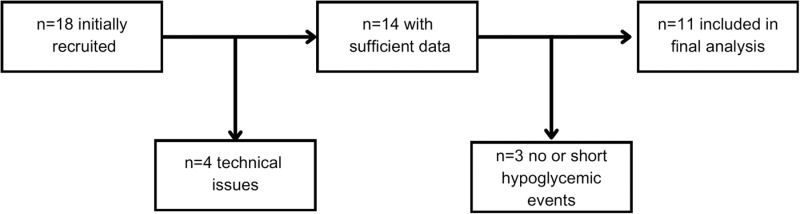
Flowchart depicting the recruitment process, showing the reasons for excluding participants at different stages. In total, 18 participants were recruited. Four individuals encountered technical issues leading to data loss, meaning there were 14 participants with sufficient data. Among these, three participants experienced no or very short (< 15 minutes) hypoglycemic events; thus, data from 11 participants was ultimately included in the final analysis.

Among the 18 recruited participants, four were excluded due to technical problems leading to data loss and three because they experienced no or very short hypoglycemic events (Fig 3). Thus, 11 participants (9 males, 2 females) were included in the final analysis, aged 39 ± 12 years (mean ± SD), weighing 85 ± 17 kg, and with a BMI of 26.2 ± 2.9, HbA1c of 7.1% ± 0.46%, and disease duration of 15.6 ± 5.5 years (Table 2).

**Table 2:** Main characteristics of the final included study population.

Characteristic	Value
Participants, n	11
Age (years), mean ± SD	39 ± 12
Weight (kg), mean ± SD	85 ± 17
BMI, mean ± SD	26.2 ± 2.9
HbA1c, mean ± SD	
%	7.1% ± 0.46
mmol/mol	54.1 ± 5
Disease duration (years), mean ± SD	15.6 ± 5.5
Sex, n	
Male	9
Female	2
Insulin treatment, n	
CSII^*^ MDI^**^	2 9

* Continuous subcutaneous insulin infusion.

** Multiple daily injection.

### Model performance

**Fig 4:**
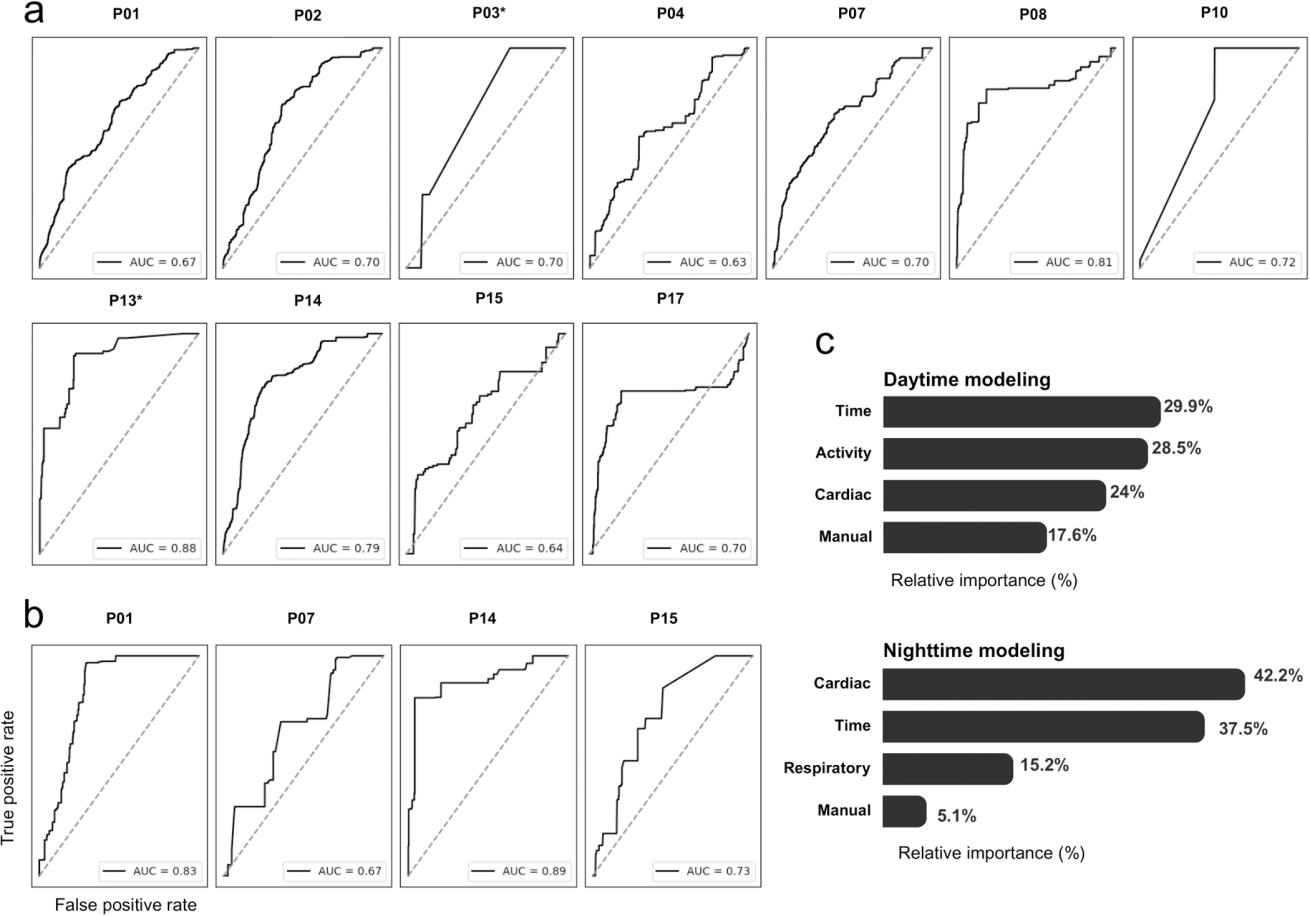
Performance of personalized models and contribution of feature categories to model predictions. a. Daytime model performance visualized via AUROC; each curve represents model performance for one participant. Participants P03 and P13 (*) were modeled without mealtime-derived features (see main text). b. Nighttime model performance. c. Average relative importance of feature categories to model predictions. “Cardiac” features comprise HR and HRV features, “Time” features are sine and cosine transformations of the hour of day, “Activity” features comprise step count and active energy expenditure features, and “Manual” features are time elapsed since the last meal and insulin on board.

While all participants experienced one or more daytime hypoglycemic events, only six participants experienced hypoglycemia at nighttime. Consequently, we report the performance of 17 personalized predictive models (11 daytime and 6 nighttime). Evaluation of model performance yielded an average AUROC score of 0.74 ± 0.08, and average specificity and sensitivity of 0.76 ± 0.18 and 0.71 ± 0.15, respectively. Individual model performances are shown in Fig 4.

Daytime modeling showed overall adequate performance for all participants, out of which two (P03 & P13) were modeled without the mealtime-derived feature due to incomplete meal records (Fig 4A). Nighttime modeling was possible for four out of six participants (Fig 4B) but could not be performed for two others who experienced inconsistent sleeping times and/or sleep restrictions due to night shifts or military service. Among modeled hypoglycemic events (n = 101), 60.4% were identified by the model at any time during the event and 36.6% were identified within the first 15 minutes.

### Model interpretability

Analysis of absolute SHAP values averaged across all participants indicated that for daytime modeling, the time-of-day, activity, and cardiac features made comparable contributions to the model’s decision (29.9%, 28.5%, and 24% contribution to the model’s decision, respectively), whereas manual features accounted for 17.6%. For nighttime models, cardiac features demonstrated the highest importance (42.2%), followed by time-of-day features (37.5%), and respiratory features (15.2%), whereas manual features showed minimal importance (Fig 3C). Table 3 details the breakdown of the results of this analysis and the average relative importance of individual features.

**Table 3:** Relative importance of feature categories and individual features averaged among participants.

Daytime models			
Feature category	Average importance (%)	Individual features	Average importance (%)
Cardiac features	24.0	Heart rate (10 min rolling average)	5.7
		Heart rate (30 min rolling average)	5.1
		Heart rate (60 min rolling average)	7.3
		Heart rate variability	4.3
		Heart rate variability (difference from previous reading)	1.6
Activity features	28.5	Step count (30 min rolling sum)	8.9
		Step count (60 min rolling sum)	10.3
		Active energy expenditure (30 min rolling sum)	3.7
		Active energy expenditure (60 min rolling sum)	5.6
Manual features	17.6	Time since last meal	11.4
		Insulin on board	6.2
Time-of-day features	29.9	Hour of the day (sine transformation)	22.1
		Hour of the day (cosine transformation)	7.9
Nighttime models			
Feature category	Average importance (%)	Individual features	Average importance (%)
Cardiac features	42.2	Heart rate (10 min rolling average)	3.5
		Heart rate (30 min rolling average)	8.4
		Heart rate (60 min rolling average)	15.3
		Heart rate variability	8.4
		Heart rate variability (difference from previous reading)	6.5
Respiratory features	15.2	Blood oxygen saturation	11.8
		Respiratory rate	3.5
Manual features	5.1	Time since last meal	3.3
		Insulin on board	1.8
Time-of-day features	37.5	Hour of the day (sine transformation)	19.7
		Hour of the day (cosine transformation)	17.8

## Discussion

In this study, we demonstrated the potential of using a consumer-grade smartwatch for noninvasive hypoglycemia detection in people with type 1 diabetes. The results showed adequate model performance (average AUROC of 0.74 ± 0.08) using 10 or 13 input features for sleeping and waking models, respectively, and support previous research by Lehman et al. [18] who reported an AUROC value of 0.76 ± 0.07 using a set of 37 features.

Our reported feature importance also aligns with prior reports. Time-of-day and cardiac features were highly important, as in previous studies [18,25]. The importance of cardiac features in both daytime and nighttime models is consistent with existing findings regarding the effects of hypoglycemia on the cardiovascular system [13,26]. The low importance of HRV features in daytime models is likely due to infrequent HRV measurements by the Apple Watch during the day, resulting in limited HRV data. This explanation is supported by the greater importance of HRV features observed in nighttime models, where measurements were made more frequently and consistently. Nighttime modeling could not be performed for two participants with sleep restrictions and/or inconsistent sleeping times. Given the strong influence of time-of-day features on the model output, it is clear that inconsistent sleep patterns impair model performance. Also, sleep restriction reportedly affects HRV [27,28], another prominent feature in model predictions. Thus, sleep quality must be considered as an important factor when detecting hypoglycemia noninvasively using consumer-grade wearables.

Two physiological parameters, RR and BOS, have not been considered in similar previous research. Our analysis revealed that these respiratory features exhibit a collective importance comparable to that of HRV in nighttime models. This finding is in agreement with reports that demonstrated that hypoglycemia leads to respiratory responses such as hyperventilation [29,30].

The importance of the additional manually-collected features, namely the time since last meal and insulin on board, was found to be the lowest in both daytime and nighttime models. It is worth noting that we included both of these features in modeling in a simplistic manner to allow for practical implementation in real-life settings (e.g., in the form of a smartwatch application, a shortcut option to quickly log a meal or insulin administration could be implemented).

Overall, the present results are promising despite the challenges of the employed real-life setting, data discontinuity from the Apple Watch, simplistic reporting of mealtimes and insulin usage, and a relatively low number of input features. Our personalized approach renders practical application feasible; modeling using the individual’s own data i) accommodates interpersonal differences in daily habits, activity levels, and ranges of physiological parameters, as well as varying physiological responses to hypoglycemia [9], ii) allows the model to be continuously retrained and updated to reflect changes in one’s lifestyle, and iii) circumvents challenges of using a shared model built on data from several individuals where continuous model calibration across geographical regions and populations is necessary for generalizability. Notably, the relative importance of input features varied between participants, which further supports this approach.

Considering the limitations of this study, we acknowledge the relatively limited number of participants that were included in the final analyses. However, the number of recorded hypoglycemic events exceeded or was comparable to that in previous studies [9,14,15]. Nevertheless, the small sample size and relatively short data collection period may limit the potential generalizability of our findings. Another aspect that limits generalizability is the inclusion criteria employed in the study, which may have introduced a selection bias. Moreover, despite a partially balanced gender distribution in the initially recruited population, a gender imbalance was present in the included population. Thus, future studies with longer duration and larger, more diverse samples are needed to replicate and confirm our findings. In addition, different smartwatch brands and models should be investigated to ensure reproducibility, due to variations in sensor technology.

Possible methods to improve future model performance include: (i) incorporating continuous data collection for most or all parameters with few or no missing data points, which would omit the need for data imputation and provide more granular data for model training; and (ii) including electrodermal activity data as an input feature, given its reported importance for hypoglycemia detection [18,31].

In conclusion, this study demonstrates the potential for noninvasive hypoglycemia detection using data from a consumer-grade wearable. The personalized methodology we employed supports practical implementation and could provide an additional health management tool for people with diabetes. In addition, the reported relative importance of input features in waking and sleeping states offers valuable insights into the most influential parameters to be considered in future research.

## Supporting information

S1 Table List of inclusion and exclusion criteria. (DOCX)
