# Supplementary material for: Personalized machine learning models for noninvasive hypoglycemia detection in people with type 1 diabetes using a smartwatch: Insights into feature importance during waking and sleeping times
Source: PLoS One. 2025 Jun 25;20(6):e0325956. doi: 10.1371/journal.pone.0325956 (PMC12193853; doi:10.1371/journal.pone.0325956)
Supplement: S1 Table — (DOCX) [file pone.0325956.s001.docx]

**Supplemental material**

**S1 Table. List of inclusion and exclusion criteria**

| Inclusion criteria | Exclusion criteria |
| --- | --- |
| • Has type 1 diabetes  • Aged between 18 and 65 years  • Can speak German  • Capable of using a smartwatch  • Uses MDI (multiple daily injections) or conventional subcutaneous insulin infusion (CSII) without a hybrid closed-loop system  • Willing to measure blood sugar by capillary measurements during the study phase (concomitant use of CGMS and flash glucose monitoring systems during the study period was not permitted)  • Diabetes duration from 1–15 years  • HbA1c between 7–10% (DCCT)  • BMI between 20 and 27 | • Use of medication affecting heart rate (i.e., beta-blockers, antiarrhythmics)  • Patients with pacemakers and implanted cardioverter-defibrillators (ICDs)  • Having other comorbidities, especially cardiovascular conditions (such as arrythmia, hypertension, and heart failure), renal insufficiency, and liver insufficiency  • Patients experiencing one or more severe hypoglycemia episodes per month  • Patients with severe hypoglycemia unawareness (Clarke score ≥4)  • Users of hybrid closed-loop systems  • Patients with high habitual alcohol consumption  • Patients who are highly athletic and regularly undergo intensive physical activity |
